# Factors Associated with the Prevalence of Dengue–Leptospirosis Coinfection in Patients Hospitalized for Febrile Syndrome

**DOI:** 10.3390/tropicalmed11020050

**Published:** 2026-02-12

**Authors:** Dina I. Bance-Anicama, María M. Diaz-Orihuela, Luz M. Diaz-Orihuela, Wilter C. Morales-García

**Affiliations:** 1 Unidad de Salud Pública, Escuela de Posgrado, Universidad Peruana Unión, Lima 15072, Peru; 2 Dirección General de Investigación e Innovación, Universidad Peruana Unión, Lima 15072, Peru

**Keywords:** dengue, leptospirosis, coinfection, febrile syndrome, integrated surveillance

## Abstract

Background: In tropical regions, dengue and leptospirosis coexist and share a nonspecific clinical onset that hinders timely diagnosis. Coinfection may worsen the clinical course and increase mortality. Objective: To estimate the prevalence of dengue, leptospirosis, and coinfection among patients with febrile syndrome in Madre de Dios (Peru) and to identify associated clinical factors. Methods: Observational, analytical, cross-sectional, retrospective study conducted at a primary-level health facility. Clinical and laboratory records of patients with febrile syndrome seen in 2024 were analyzed. Categorical variables were summarized as frequencies (%) and numeric variables as mean ± SD or median [IQR]. Comparisons used chi-square or Fisher’s exact test, Student’s *t* test, or the Mann–Whitney U test, as appropriate. Associations were estimated using Poisson regression models with robust variance, adjusted for sex, reporting prevalence ratios (PRs) and 95% CIs. Analyses were performed in R 4.0.2. Results: A total of 226 patients were included. Positivity was 19.0% for dengue (43/226), 66.8% for leptospirosis (151/226), and 5.8% for coinfection (13/226). In the bivariate analysis, dengue was associated with higher temperature (*p* < 0.001), lower mean arterial pressure (*p* = 0.007), mucosal bleeding/ecchymosis (*p* = 0.049), and lower fluid intake (*p* = 0.021); temperature was also higher in coinfection (*p* = 0.021). In Poisson models, dengue was associated with tachycardia (PR = 5.69; 95% CI: 1.95–13.07; *p* < 0.001), temperature (PR = 1.61 per °C; 1.23–2.12; *p* = 0.001), bilateral polyarthralgia (PR = 2.55; 1.14–5.04; *p* = 0.012), and mucosal bleeding/ecchymosis (PR = 3.31; 0.94–8.37; *p* = 0.027). Leptospirosis was associated with male sex (PR = 0.78 vs. female; 0.65–0.94; *p* = 0.010) and fever (PR = 2.38; 1.17–6.03; *p* = 0.035). Leptospira–dengue coinfection was related to higher temperature (PR = 1.75 per °C; 1.05–3.01; *p* = 0.036). Conclusions: Simple clinical signs such as fever/elevated temperature, tachycardia, bilateral polyarthralgia, and mucosal bleeding can help prioritize suspicion of dengue, leptospirosis, or coinfection; guide requests for dual testing (dengue–Leptospira), early hydration in dengue, and timely initiation of antibiotic therapy in leptospirosis. These findings support the development of integrated triage algorithms and strengthening access to molecular diagnostics in high-burden febrile syndrome settings.

## 1. Introduction

Global dengue incidence has increased substantially over the past decades; the WHO documented that the number of reported cases worldwide had increased tenfold [1]. In 2023, 4.1 million suspected dengue cases were reported, including 6710 severe cases and 2049 deaths across 42 countries and territories in the Region of the Americas, and 15 countries reported an active outbreak [2]. In contrast, leptospirosis has historically been underreported in the Region of the Americas. Early regional analyses indicate that several tropical countries, including Brazil, Colombia, and Peru, report meaningful annual incidence and outbreaks associated with flooding and natural disasters, positioning leptospirosis as a priority zoonosis in the region [3]. In Peru, 1193 leptospirosis cases were reported from 2019 to 2024, and 97.49% of these were classified as probable cases [4].

Tropical and subtropical regions face a disproportionate burden of emerging and re-emerging infectious diseases. Among these, dengue and leptospirosis are priority conditions due to their high incidence, morbidity, and potential lethality [5]. The two diseases not only overlap in endemic geographic areas but also share similar initial clinical features and environmental conditions that facilitate their joint spread [6]. This overlap has sparked growing scientific interest in understanding the magnitude, associated factors, and clinical implications of dengue leptospirosis coinfection, an underestimated yet clinically relevant entity [7].

Dengue is a viral disease transmitted by mosquitoes of the *Aedes* genus—primarily *Aedes aegypti*—and is the most prevalent arboviral disease worldwide. Its estimated burden is 390 million infections annually, of which 100 million develop clinical manifestations, and up to 40,000 people die from severe dengue. Its high frequency is linked to environmental factors such as warm, humid climates, the accumulation of stagnant water, high urban population density, and inadequate basic sanitation, as well as social and behavioral factors such as lack of personal protective measures, poor solid-waste and water management, and limited public knowledge about prevention [8]. Leptospirosis, by contrast, is a bacterial zoonosis caused by spirochetes of the genus *Leptospira* that enter the body through broken skin (including cuts) and mucous membranes such as the eyes, nose, and mouth. This occurs because animal reservoirs—such as rodents, dogs, cattle, horses, pigs, goats, and wildlife—shed the bacteria in their urine, contaminating the environment. Transmission occurs through contact with water, soil, or food contaminated with infected urine. Its high frequency is associated with risk activities such as agriculture, mining, and sewer work, as well as natural disasters; environmental factors such as flooding, heavy rainfall, tropical climates, poor waste management, and rodent presence; and social determinants such as limited access to safe drinking water, lack of basic sanitation, homes located near stagnant water or dumpsites, and limited knowledge about prevention. Globally, leptospirosis is estimated to cause more than 1 million human cases each year and around 60,000 deaths, primarily due to severe forms such as Weil’s syndrome and massive pulmonary hemorrhage, with case-fatality rates that can reach 5–15% in resource-limited settings [1,9,10]. Therefore, when it is stated that “this disease affects more than one million people per year,” this refers to the global burden of leptospirosis, comparable in magnitude to other priority tropical febrile illnesses.

Despite their distinct etiologies, one viral and the other bacterial, both diseases share a similar initial clinical presentation: fever, myalgia, headache, general malaise, gastrointestinal symptoms, and even hemorrhagic or respiratory manifestations in severe phases [1,11]. This similarity represents a clinical barrier to differential diagnosis, particularly in epidemic contexts or in resource-limited areas; indeed, numerous studies have documented that a substantial proportion of patients initially diagnosed with dengue and who test negative for this virus are later positive for leptospirosis, revealing an underestimated burden of the latter [12].

In this context, dengue–leptospirosis coinfection emerges as a concerning clinical entity; both diseases can coincide not only in time and space but also in the same individual, producing more severe clinical pictures and potential complications such as renal failure, massive pulmonary hemorrhage, shock syndrome, or multiorgan failure [13,14]. Therapeutic management becomes more complex because differentiated interventions are required: while dengue calls for hemodynamic monitoring and symptomatic management, leptospirosis requires early antibiotic therapy to prevent serious sequelae such as Weil’s syndrome [13].

Environmental factors such as heavy rainfall, flooding, and the El Niño phenomenon have been widely linked to concurrent increases in both pathogens. These conditions foster the breeding of the dengue mosquito vector and increase exposure to animal reservoirs and environments contaminated with *Leptospira* [15,16,17]. In addition, socioeconomic factors such as unplanned urbanization, poverty, limited access to basic services, and overcrowding further heighten the vulnerability of affected populations [18]. Previous studies examining the possible coexistence of dengue and leptospirosis can be grouped into two main categories. First are investigations conducted in cohorts of patients with “clinically suspected dengue” in which only leptospirosis was confirmed. In these settings, *Leptospira* positivity among dengue-negative cases suggests misdiagnosis or simultaneous circulation of both pathogens, but it does not demonstrate coinfection within the same clinical episode. For example, in Yucatán, Mexico, 14% of clinically diagnosed dengue cases tested positive for leptospirosis by the MAT [19]. Similarly, during a dengue outbreak in Bangladesh, LaRocque et al. identified confirmed leptospirosis in patients with acute fever initially suspected to be dengue but with negative dengue tests, demonstrating the presence of leptospirosis in the context of a dengue outbreak without documenting laboratory-confirmed coinfections [20]. Documented reports from Paraguay (8%), Jamaica (5%), Veracruz (12%), and the Brazilian Amazon point to an emerging burden in areas with high rainfall and limited public health infrastructure [21,22,23,24].

Second, there is a much smaller body of studies in which patients were tested simultaneously for dengue and leptospirosis using laboratory methods, allowing true coinfections to be demonstrated. For instance, in a hospital-based study in Malaysia, Suppiah et al. [25] identified laboratory-confirmed dengue–leptospirosis coinfection in approximately 4% of patients admitted with dengue and described specific clinical predictors such as jaundice, acute kidney injury, and more severe thrombocytopenia. However, this type of research remains scarce, and most available reports are case series or small-sample studies, limiting precise estimation of the true prevalence of coinfections.

Coinfection prevalence varies not only by geography but also by the availability of appropriate diagnostic tools. Limited sensitivity of serologic tests in early stages, lack of access to PCR in many health centers, and reliance on nonspecific clinical algorithms all hinder accurate estimation of the true burden of coinfections [26,27]. These diagnostic constraints contribute to substantial underreporting and delays in initiating appropriate treatment, particularly for leptospirosis, where the early therapeutic window is critical to preventing severe complications [28].

Coinfection occurs more frequently in young adults, predominantly men, and has been reported more often in peri-urban communities, riverine areas, and rural settings with limited access to safe water and sanitation [12,29]. In a study in São Paulo, Brazil, higher leptospirosis seroprevalence was observed in men (1.56%) than in women (0.56%), suggesting differential exposure likely related to occupational or recreational practices [12]. Beyond its clinical impact, coinfection poses a public health challenge: lack of awareness of its coexistence can lead to diagnostic errors, inappropriate treatment, and increased mortality.

In Puerto Rico, deaths associated with dengue–leptospirosis coinfection were documented, underscoring the synergistic effect of both conditions when they are not correctly identified and treated [30]. In Malaysia, a study of 321 febrile patients reported that, among dengue-negative cases, more than 20% were positive for leptospirosis—a substantial proportion that highlights the need for dual clinical suspicion [11]. The situation is further complicated when other febrile illnesses such as malaria, chikungunya, yellow fever, typhoid fever, or rickettsioses are also prevalent in the same regions, competing in the differential diagnosis. In addition, concomitant leptospirosis coinfections with other emerging viral pathogens, such as hantaviruses, have been described in patients hospitalized with acute febrile syndrome, pointing to a potentially underestimated global problem and underscoring the need for multi-etiologic diagnostic approaches [31]. In India, typhus–leptospirosis coinfection was more frequent (67.44%) than dengue–leptospirosis coinfection, reinforcing the need to implement molecular testing and multi-etiologic clinical algorithms [32].

In Peru, a recent study found a 5.9% prevalence of coinfection among febrile patients with suspected dengue, along with 11.2% of pure leptospirosis cases [33]. Another study in Madre de Dios showed a high incidence of fever as a predictive symptom of dengue but also acknowledged the possibility of multiple etiologies, including leptospirosis [34]. In response to this scenario, Peru’s Ministry of Health has prioritized the implementation of febrile units and the use of epidemiologic forms for simultaneous screening of dengue, leptospirosis, malaria, and other arboviruses. From a regulatory standpoint, Peru and other Latin American countries have issued separate clinical guidelines for dengue and leptospirosis management [35,36]. Within this context of high disease burden, complex diagnosis, and still-limited evidence on laboratory-confirmed coinfections, the present study aims to assess factors associated with the prevalence of dengue and leptospirosis coinfection among patients with febrile syndrome treated at a health facility in the department of Madre de Dios, Peru.

## 2. Methods

### 2.1. Design and Participants

The present study used an observational, analytical, cross-sectional, retrospective design [37]. The minimum sample size was 175 participants, estimated using G*Power 3.1 for a two-sided binary logistic regression model, assuming an odds ratio (OR) of 4.5, an expected event prevalence (coinfection) of 5%, a 5% significance level (α = 0.05), and 80% statistical power. Nevertheless, all records meeting the inclusion criteria were analyzed, yielding a final sample of 226 patients. A nonprobability convenience sampling approach was used. Participants were drawn from a public primary care health facility (category I-2) located in the department of Madre de Dios, Peru. Patients were included if they presented with febrile syndrome during 2024, regardless of sex, age, or place of origin and had testing ordered to rule out dengue and leptospirosis, as well as a complete blood count; all were monitored using the standardized follow-up form until medical discharge. Febrile syndrome was defined as an axillary temperature ≥ 38 °C or a history of fever lasting up to 7 days, accompanied by at least one nonspecific symptom (headache, myalgia, arthralgia, or malaise), in accordance with the National Technical Standard [38]. Patients who did not meet these criteria were excluded. The analysis considered three mutually exclusive diagnostic categories: confirmed dengue, confirmed leptospirosis, and coinfection (dengue + *Leptospira*). Patients with negative tests for both pathogens were retained in the study as febrile syndrome cases without etiologic confirmation (“double negatives”) and served as the comparison group rather than healthy individuals.

#### Instruments

Institutional clinical records from 2024 at the I-2 health facility were used. These included the Febrile Unit logbooks; laboratory results for dengue and Leptospira diagnosis recorded in the national NetLab system; signs and symptoms documented in the dengue and leptospirosis clinical–epidemiologic form; complete blood count results recorded in the facility’s laboratory logbook; and sociodemographic data, as well as information on the staff conducting monitoring, recorded on the monitoring form established by the Ministry of Health Clinical Practice Guideline for dengue and leptospirosis care [38]. To preserve confidentiality, no direct identifiers were collected, and potentially identifiable variables were grouped when appropriate. For dengue diagnosis, a confirmed case was defined as having at least one positive NS1 antigen detection test by immunochromatography or ELISA within the first five days of illness and/or dengue-specific IgM by ELISA from day five onward, following the cutoffs established in the National Dengue Technical Standard [38]. For leptospirosis, a confirmed case was defined as a reactive *Leptospira* spp. IgM ELISA or a reactive microscopic agglutination test (MAT) according to national criteria; cases with both tests negative were classified as unconfirmed. Coinfection cases were defined by the simultaneous presence of at least one positive dengue test and one positive leptospirosis test during the same febrile syndrome episode.

### 2.2. Ethical Aspects

The research adhered strictly to the principles of justice, beneficence, non-maleficence, and respect for autonomy; the Declaration of Helsinki; the Technical Health Standard for health research of the Ministry of Health of Peru; and the guidelines of the Council for International Organizations of Medical Sciences (CIOMS). Given the retrospective nature and the use of institutional records, no direct interventions were performed and clinical variables were not altered. Data were pseudonymized prior to analysis; no direct identifiers were included, and grouping strategies were implemented to reduce the risk of reidentification. The protocol was approved by the Research Ethics Committee of the Graduate School of the Universidad Peruana Unión (code 2025-CE-EPG-00019), ensuring confidentiality, anonymity, and protection of participant identity.

### 2.3. Data Analysis

Variables were described according to their nature: categorical variables were presented as absolute and relative frequencies (%), whereas numeric variables were summarized using the mean and standard deviation (SD) or the median and interquartile range (IQR), depending on the data distribution. Normality of numeric variables was assessed using the Kolmogorov–Smirnov test. For between-group comparisons, statistical tests appropriate to the data type were used: the chi-square test or Fisher’s exact test for categorical variables, and Student’s *t* test or the Mann–Whitney U test for numeric variables, as appropriate. To identify associations between the factors evaluated and dengue virus infection, leptospirosis, or coinfection, Poisson regression models with robust variance were fitted for each binary outcome (dengue yes/no, leptospirosis yes/no, coinfection yes/no), estimating prevalence ratios (PRs) with their corresponding 95% confidence intervals (95% CIs). Given the sample size and the low frequency of coinfection, models were adjusted for sex as a control covariate. In all analyses, a *p* value < 0.05 was considered statistically significant.

Statistical analyses were performed using the R programming language, version 4.0.2 (R Foundation for Statistical Computing, Austria, Vienna).

## 3. Results

### 3.1. Bivariate Analysis

Table 1, which presents sociodemographic variables, general clinical characteristics, and vital signs of patients with leptospirosis, dengue, and coinfection, shows that most comparisons did not reach statistical significance, except for several key indicators. In the leptospirosis group, sex showed a significant difference (*p* = 0.014), with a higher proportion of women among those infected (74.6%) compared to those not infected (25.4%). Among patients with dengue, statistically significant differences were observed in several vital signs and clinical symptoms: mean arterial pressure (MAP) was lower in positive cases (70.0 mmHg) compared to negatives (77.7 mmHg) (*p* = 0.007); body temperature was higher in patients with dengue (*p* < 0.001), and was also significantly higher in cases of coinfection (*p* = 0.021). In addition, mucosal bleeding or ecchymosis was significantly associated with a dengue diagnosis (*p* = 0.049). Other symptoms, such as persistent vomiting, chest pain, somnolence and other vital signs (pulse, oxygen saturation, respiratory rate) did not show statistically significant differences between groups. These findings suggest that certain clinical features, such as sex, MAP, and temperature, may be useful as differential indicators, particularly for the diagnosis of dengue or coinfection.

Table 2 describes additional clinical signs, laboratory tests, and follow-up–related variables. The amount of fluids consumed in the previous 24 h was significantly associated with a diagnosis of dengue (*p* = 0.021) and coinfection (*p* = 0.014); patients with dengue and, to an even greater extent, those with coinfection reported lower fluid intake, suggesting a higher risk of dehydration in these groups. Regarding follow-up, among patients with dengue, monitoring was performed predominantly by nursing staff, with significant differences by type of professional responsible for follow-up (*p* = 0.047). Likewise, the presence of bilateral polyarthralgia was significantly associated with a dengue diagnosis (*p* = 0.017). For leptospirosis, an association with female sex was confirmed (*p* = 0.014), consistent with what was observed in Table 1, and the presence of fever showed a significant relationship with a leptospirosis diagnosis (*p* = 0.042). Laboratory variables (hemoglobin, hematocrit, platelets, and leukocytes) did not show statistically significant differences across groups for leptospirosis, dengue, or coinfection. With respect to the time elapsed between symptom onset and medical care, the median “delay to care (days)” was 2 days (IQR 1–4) across all diagnostic groups, with no significant differences for leptospirosis (*p* = 0.745), dengue (*p* = 0.265), or coinfection (*p* = 0.869). This indicates that temperature measurements and fever assessment were generally performed within a similar time window after onset of the febrile illness across groups.

### 3.2. Factors Associated with Dengue, Leptospirosis, and Coinfection

Results from Table 3 show that several clinical variables are significantly associated with the presence of dengue, leptospirosis, or coinfection. In cases of *Leptospira*–dengue coinfection, body temperature was significantly associated with higher prevalence, with a crude prevalence ratio (cPR) of 1.75 (95% CI: 1.05–3.01; *p* = 0.036), indicating that for each 1 °C increase in temperature, the risk of coinfection rises by approximately 75%. Among patients with dengue, the main associated clinical factors were tachycardia, with a cPR of 5.69 (95% CI: 1.95–13.07; *p* < 0.001), representing nearly a sixfold higher likelihood of dengue in the presence of this sign; elevated body temperature (cPR = 1.61; 95% CI: 1.23–2.12; *p* = 0.001); bilateral polyarthralgia (cPR = 2.55; 95% CI: 1.14–5.04; *p* = 0.012); and mucosal bleeding or ecchymosis (cPR = 3.31; 95% CI: 0.94–8.37; *p* = 0.027), the latter showing a borderline association. For *Leptospira* infection, the significantly associated factors were male sex, with a lower probability of leptospirosis compared with females (cPR = 0.78; 95% CI: 0.65–0.94; *p* = 0.010), and the presence of fever, which more than doubled the likelihood of infection (cPR = 2.38; 95% CI: 1.17–6.03; *p* = 0.035). Taken together, these findings underscore the importance of clinical signs such as fever, tachycardia, elevated temperature, and bilateral polyarthralgia as key predictors in the differential diagnosis of dengue, leptospirosis, and their coinfection, within a relatively homogeneous time window from symptom onset.

## 4. Discussion

The burden of dengue has increased markedly worldwide and in the Americas, while leptospirosis persists in tropical settings. The two diseases share endemic areas, environmental drivers, and a similar initial clinical presentation, which hinders differential diagnosis and favors underreporting. Dengue–leptospirosis coinfection, although often overlooked, is associated with greater severity (renal failure, pulmonary hemorrhage, shock) and requires distinct therapeutic approaches (supportive care in dengue, early antibiotic therapy in leptospirosis). Rainfall, flooding, and El Niño, together with unplanned urbanization, poverty, and sanitation deficiencies, increase risk. Limited availability of sensitive tests further delays diagnosis. In Peru, coinfection is documented, and MINSA promotes febrile units and forms for multiple exclusion testing. Therefore, the objective of this study was to estimate the prevalence of dengue–leptospirosis coinfection and to determine its associated factors in patients with febrile syndrome treated at a health facility in Madre de Dios, Peru.

Statistically significant sex differences were observed between patients with and without leptospirosis. The proportion of women with leptospirosis was higher, which may reflect greater differential exposure rather than biological susceptibility: domestic tasks involving food handling, contact with stagnant water, domestic animals, and sanitation deficiencies could increase risk. Similar findings were reported in Ayacucho and Madre de Dios, where women accounted for more infections and linked occupations were agriculture, housework, studying, and mining [34,39]. However, other contexts show the opposite pattern. In northern Peru, there was a slight male predominance, associated with contact with animals and occupations such as student, housework, agriculture, and livestock [40]. In another tropical region of the country, males also predominated, with rice farmers as the main group [41]. In Malaysia, greater impact was likewise described among men engaged in high-risk outdoor occupations (agriculture, military, healthcare personnel, field work) [42]. Taken together, the higher positivity in men described in various studies appears to reflect a combination of occupational, behavioral, and socioeconomic factors as well as frequency of exposure to potentially contaminated water sources, rather than intrinsic differences in susceptibility [39].

Patients with dengue had significantly lower mean arterial pressure (MAP) values than those without dengue, whose readings remained within normal ranges. This finding suggests early hemodynamic compromise in dengue and could be useful for triage, close monitoring, and timely initiation of interventions. Although comorbidities such as diabetes and hypertension increase the risk of severe outcomes, hypotension in dengue is itself a severity criterion, attributed to plasma leakage due to increased capillary permeability, reduced venous return, and inflammation-mediated vasodilation, in addition to febrile dehydration and, when present, blood loss. These mechanisms support the association between dengue and low MAP described in the literature [34,38,39,40,41,42].

Likewise, among patients with *Leptospira*–dengue coinfection, body temperature was significantly higher than in those without coinfection; most presented with low-grade fever or fever. This difference was also observed in the isolated dengue group, reinforcing fever as a distinctive clinical sign. The literature attributes this rise in temperature to an exaggerated inflammatory response, with immune hyperstimulation and potential tissue damage proportional to disease severity [1,34,38,42]. Fever was significantly associated with *Leptospira*–dengue coinfection, so in co-circulation settings it could be considered an early clinical marker. However, it is important to recognize that fever and temperature depend largely on the stage of illness. In our sample, the median number of days between symptom onset and medical care was 2 days across all diagnostic groups, with no significant differences, suggesting that assessment occurred within a relatively homogeneous time window. Even so, individual variability in “day of illness” at the time of measurement may have influenced the strength of the association between temperature and coinfection; therefore, these results should be interpreted with caution.

Acute febrile syndrome is a common reason for emergency visits; although many cases do not require hospitalization, the presence of fever more than doubled the risk of leptospirosis in our analysis, confirming its value as a cardinal symptom. In turn, male sex showed a lower likelihood of diagnosis compared with female sex, possibly reflecting differences in environmental exposure, access to care, or immune response. Clinical evolution should be monitored for signs of meningitis, jaundice with or without hepatorenal involvement, massive pulmonary hemorrhage, or Weil’s syndrome. While some reports describe higher frequency in men (with more severe forms or higher case fatality), others find a female predominance; in both cases, social and occupational factors play an important role. These findings support strengthening prevention, timely diagnosis, and treatment strategies [1,7,43,44].

Regarding mucosal bleeding/ecchymosis, although this variable did not reach significance in the leptospirosis group, a significant difference was observed in the dengue group, where mucosal bleeding was more frequent. This finding suggests diagnostic utility in more severe phases and supports clinical classification toward dengue with warning signs or severe dengue; without close monitoring and timely intervention, potentially fatal complications may occur [1,38,42]. A study in Colombia on factors associated with severe dengue reported a high proportion of cases and identified the following predictors: days elapsed before seeking care, hypotension, hepatomegaly, mucosal hemorrhage, thrombocytopenia < 100,000, and fluid accumulation—all warning signs that justify hospitalization [45].

In our analysis, tachycardia emerged as the strongest predictor, quintupling the risk of dengue and occurring alongside elevated temperature. Bilateral polyarthralgia and mucosal bleeding/ecchymosis also showed significant associations. These findings are consistent with the literature describing combined systemic effects (endothelial injury, intense inflammation, and coagulation abnormalities) that lead to hemodynamic instability [29,46,47,48]. Finally, community risk for dengue also depends on population knowledge, attitudes, and practices. Exposure is linked to behaviors such as water storage, plant care, and personal protection against mosquito bites. Systematic vector surveillance and community-based control actions substantially increase resilience to outbreaks [10].

### 4.1. Implications

The results reveal a clinical pattern that may be useful for decision-making in resource-limited settings with a high burden of febrile syndromes. In clinical practice, the combination of fever/elevated temperature and tachycardia could substantially increase the likelihood of dengue, whereas isolated fever and female sex were associated with leptospirosis; additionally, increases in temperature were linked to a higher prevalence of coinfection. Given the small sample size of the coinfection group, these associations should be viewed as exploratory findings rather than definitive decision rules.

These findings suggest the potential to develop stepwise triage protocols: (1) early dual suspicion in the presence of high fever with warning signs (tachycardia, mucosal bleeding/ecchymosis, bilateral polyarthralgia), prioritizing simultaneous testing for dengue and *Leptospira*; (2) early hydration and close monitoring in dengue, as the lower fluid intake during the first days identified in this study reinforces the need to standardize minimum intake volumes and track fluid balance; and (3) early initiation of antibiotics in patients with moderate-to-high clinical probability of leptospirosis, in line with national guidelines, avoiding delays that increase the risk of complications. The most complex scenario is a febrile patient with low initial clinical probability of leptospirosis and suspected dengue or coinfection not yet confirmed within the first 24 h; in such cases, empiric antibiotic treatment could be considered until confirmatory results are available, especially if clinical suspicion persists despite initially negative tests, always weighing the individual risk–benefit balance.

Operationally, it would be advisable to standardize clinical pathways for “undifferentiated fever” that include hospitalization criteria, testing algorithms (serology/PCR according to day of illness), and clearly defined roles for nursing teams (who conducted most of the monitoring in our setting), supported by vital-sign checklists and warning-sign alerts.

From a public health perspective, these findings could support integrated control strategies that combine *Aedes* vector management with reservoir control and sanitation measures targeting *Leptospira*, particularly during rainy and flood seasons. Rather than issuing rigid recommendations, our results suggest the value of strengthening dual syndromic screening in Febrile Units, ensuring continuous availability of diagnostic supplies and first-line antibiotics, and expanding molecular diagnostic capacity (PCR) within regional networks to improve timeliness of diagnosis. Integrated surveillance (dengue–leptospirosis–other febrile illnesses) should be linked to information systems such as NetLab with mandatory fields for symptom onset date, specimen collection day, and test type, enabling valid estimates by diagnostic window. At the policy level, these findings point to the need to consider: (a) national coinfection protocols with criteria for suspicion, testing, and treatment; (b) performance indicators (e.g., time to dual testing, antibiotic initiation within <24–48 h in probable leptospirosis); and (c) combined community education campaigns (hydration, breeding-site control, waste management, occupational protection for high-risk tasks, safe water storage).

At the theoretical level, the data support an eco-syndromic model in which climatic, environmental, and socioeconomic factors modulate the shared risk of dengue and leptospirosis, and in which simple clinical biomarkers (temperature, tachycardia, mucosal bleeding, polyarthralgia) can serve as sentinel variables to prioritize tests and allocate resources. Future lines of inquiry include: (i) calibrated clinical prediction rules for coinfection; (ii) geospatial models integrating rainfall, flooding, and vector/reservoir density; and (iii) population precision-medicine frameworks that weigh sex, occupation, and environmental exposure.

### 4.2. Limitations

This study is observational, cross-sectional, and retrospective, with convenience sampling at a single primary-level facility; therefore, causal inference is limited and generalizability to other levels of care or regions should be made cautiously. The low prevalence of coinfection (n = 13) reduces power to detect associations and yields wide confidence intervals, which may under- or overestimate effects. Consequently, the clinical implications derived from the coinfection findings should be considered primarily hypothesis-generating.

Diagnostic classification may be affected by predominant reliance on serology and variation in the day of sample collection; without systematic PCR testing, some early leptospirosis or dengue cases may have gone undetected. Although we had a proxy for time since illness onset through the variable “delay to care (days),” the exact day of the clinical course on which fever and other vital signs were measured was not recorded in a standardized manner. This limits our ability to assess precisely how timing of presentation modulates the association between temperature, fever, and coinfection.

Measurement of symptoms/signs was based on routine clinical records and is susceptible to heterogeneity in documentation (e.g., definition of bilateral polyarthralgia, quantification of fluid intake), introducing information bias. Model adjustment was limited (adjusted for sex), so residual confounding cannot be ruled out (comorbidities, day of illness, occupational exposure, prior dengue history, home environmental conditions). Multiple comparisons in the bivariate analysis increase the risk of type I error. Finally, environmental and structural variables (local precipitation, discrete flooding events, vector density, rodent presence, access to safe water) were not incorporated, which constrains the eco-epidemiologic interpretation of the results.

To mitigate these limitations in future research, we recommend: (1) prospective cohorts with consecutive recruitment and sample-size calculations specific to coinfection; (2) standardized diagnostic protocols by day of illness combining serology with a multipathogen PCR panel; (3) collection of georeferenced environmental and social determinants; (4) broader multivariable adjustment (comorbidity, occupational exposure, time to consultation, hydration/fluid balance, severity metrics); (5) analyses using robust methods to control for multiplicity and internal/external model validation; and (6) clinical data-quality audits with unified operational definitions for signs/symptoms.

## 5. Conclusions

In a high-burden febrile syndrome setting, we found a dengue–leptospirosis coinfection prevalence close to 6%, comparable to what has previously been reported among febrile patients in Peru (around 5.9%) and similar to that described in other contexts such as Jamaica (~5%), but lower than the higher prevalences reported in Bangladesh (30%) or Yucatán, Mexico (14%). These differences suggest that the magnitude of the problem varies by ecological context, the intensity of circulation of both agents, and the availability of appropriate diagnostic testing.

Within this context, our findings point to simple, potentially actionable clinical signals to guide differential diagnosis and early management: tachycardia and elevated temperature were strongly associated with dengue; fever and female sex were linked to leptospirosis; and temperature was associated with coinfection. The lower fluid intake observed in dengue underscores the urgency of hydration and monitoring protocols from the first point of contact. Nevertheless, the small number of coinfection cases and the design limitations require cautious interpretation, and these associations should be viewed primarily as hypotheses that need confirmation in larger prospective studies.

In practice and policy, these results could support the development of dual-testing algorithms, strategies for early antibiotic initiation when leptospirosis suspicion is moderate to high, and integrated surveillance schemes within Febrile Units, as well as intersectoral interventions focused on water, sanitation, vector control, and reservoir control. Theoretically, they reinforce an eco-syndromic framework in which climatic and social determinants interact with clinical biomarkers to shape individual and population risk, and they highlight the need for new research integrating clinical, environmental, and laboratory data to better understand the dynamics of dengue–leptospirosis coinfection.

## Figures and Tables

**Table 1:** Sociodemographic variables, general clinical characteristics, and vital signs in patients with Leptospirosis, Dengue, and Coinfection.

Variable	Leptospirosis		*p*-Value	Dengue		*p*-Value	Coinfection		*p*-Value
	No (n = 75)	Yes (n = 151)		No (n = 183)	Yes (n = 43)		No (n = 213)	Yes (n = 13)	
Age (years)	32.0 [13.0–42.0]	30.0 [17.0–41.0]	0.799	31.0 [17.0–41.0]	31.0 [11.0–41.0]	0.402	31.0 [16.0–41.0]	16.0 [12.0–33.0]	0.288
Sex (%)			0.014 *			0.18			0.869
Female	30 (25.4%)	88 (74.6%)		100 (84.7%)	18 (15.3%)		112 (94.9%)	6 (5.08%)	
Male	45 (41.7%)	63 (58.3%)		83 (76.9%)	25 (23.1%)		101 (93.5%)	7 (6.48%)	
Abdominalpain (%)			0.376			0.382			0.762
No	47 (30.9%)	105 (69.1%)		126 (82.9%)	26 (17.1%)		144 (94.7%)	8 (5.26%)	
Yes	28 (37.8%)	46 (62.2%)		57 (77.0%)	17 (23.0%)		69 (93.2%)	5 (6.76%)	
Persistentvomiting (%)			0.118			0.693			0.132
No	57 (30.6%)	129 (69.4%)		152 (81.7%)	34 (18.3%)		173 (93.0%)	13 (6.99%)	
Yes	18 (45.0%)	22 (55.0%)		31 (77.5%)	9 (22.5%)		40 (100%)	0 (0.00%)	
Chestpain/shortnessofbreath (%)			0.451			0.064			0.502
No	55 (31.6%)	119 (68.4%)		146 (83.9%)	28 (16.1%)		165 (94.8%)	9 (5.17%)	
Yes	20 (38.5%)	32 (61.5%)		37 (71.2%)	15 (28.8%)		48 (92.3%)	4 (7.69%)	
Hypothermia (%)			0.109			0.345			1
No	73 (32.6%)	151 (67.4%)		182 (81.2%)	42 (18.8%)		211 (94.2%)	13 (5.80%)	
Yes	2 (100%)	0 (0.00%)		1 (50.0%)	1 (50.0%)		2 (100%)	0 (0.00%)	
Mucosalbleeding/ Ecchymosis (%)			0.336			0.049			1
No	72 (32.6%)	149 (67.4%)		181 (81.9%)	40 (18.1%)		208 (94.1%)	13 (5.88%)	
Yes	3 (60.0%)	2 (40.0%)		2 (40.0%)	3 (60.0%)		5 (100%)	0 (0.00%)	
Syncope (%)			1			0.649			0.382
No	73 (33.5%)	145 (66.5%)		177 (81.2%)	41 (18.8%)		206 (94.5%)	12 (5.50%)	
Yes	2 (25.0%)	6 (75.0%)		6 (75.0%)	2 (25.0%)		7 (87.5%)	1 (12.5%)	
Jaundice (%)			1			1			1
No	73 (33.2%)	147 (66.8%)		178 (80.9%)	42 (19.1%)		207 (94.1%)	13 (5.91%)	
Yes	2 (33.3%)	4 (66.7%)		5 (83.3%)	1 (16.7%)		6 (100%)	0 (0.00%)	
Markedsomnolence (%)			0.155			0.129			0.239
No	38 (29.0%)	93 (71.0%)		111 (84.7%)	20 (15.3%)		126 (96.2%)	5 (3.82%)	
Yes	37 (38.9%)	58 (61.1%)		72 (75.8%)	23 (24.2%)		87 (91.6%)	8 (8.42%)	
Serouseffusion (%)			1			1			1
No	74 (33.2%)	149 (66.8%)		180 (80.7%)	43 (19.3%)		210 (94.2%)	13 (5.83%)	
Yes	1 (33.3%)	2 (66.7%)		3 (100%)	0 (0.00%)		3 (100%)	0 (0.00%)	
MAP (mmHg)	74.7 [70.0–83.3]	77.7 [70.0–86.7]	0.074	77.7 [70.0–86.7]	70.0 [70.0–81.8]	0.007 **	77.0 [70.0–86.3]	72.3 [70.0–81.0]	0.219
Pulse (beats/min)	88.0 [80.0–100]	88.0 [80.0–104]	0.973	88.0 [80.0–104]	88.0 [84.0–98.0]	0.971	88.0 [80.0–103]	88.0 [84.0–99.0]	0.723
Oxygen saturation (%)	98.0 [98.0–98.0]	98.0 [98.0–98.0]	0.777	98.0 [98.0–98.0]	98.0 [98.0–98.5]	0.214	98.0 [98.0–98.0]	98.0 [98.0–99.0]	0.09
Respiratoryrate (breaths/min)	20.0 [20.0–20.0]	20.0 [20.0–20.0]	0.937	20.0 [20.0–20.0]	20.0 [18.0–20.0]	0.1	20.0 [20.0–20.0]	20.0 [18.0–22.0]	0.472
Temperature (°C)	38.0 [37.0–38.5]	37.6 [36.6–38.2]	0.23	37.6 [36.6–38.2]	38.4 [37.6–38.8]	<0.001 **	37.7 [36.6–38.3]	38.4 [38.0–38.7]	0.021 *

* *p* < 0.05; ** *p* < 0.01

**Table 2:** Complementary clinical signs, laboratory tests, and medical follow-up.

**Variable**	**Leptospirosis**		***p*** **-Value**	**Dengue**		***p*** **-Value**	**Coinfection**		***p*** **-Value**
	**No (n = 75)**	**Yes (n = 151)**		**No (n = 183)**	**Yes (n = 43)**		**No (n = 213)**	**Yes (n = 13)**	
Dry oral mucosa (%)			1			0.245			0.069
No	48 (32.9%)	98 (67.1%)		122 (83.6%)	24 (16.4%)		141 (96.6%)	5 (3.42%)	
Yes	27 (33.8%)	53 (66.2%)		61 (76.2%)	19 (23.8%)		72 (90.0%)	8 (10.0%)	
Skin turgor (pinch sign) (%)			0.688			1			0.343
No	72 (32.9%)	147 (67.1%)		177 (80.8%)	42 (19.2%)		207 (94.5%)	12 (5.48%)	
Yes	3 (42.9%)	4 (57.1%)		6 (85.7%)	1 (14.3%)		6 (85.7%)	1 (14.3%)	
Marked thirst (%)			0.334			0.971			0.774
No	44 (30.6%)	100 (69.4%)		116 (80.6%)	28 (19.4%)		135 (93.8%)	9 (6.25%)	
Yes	31 (37.8%)	51 (62.2%)		67 (81.7%)	15 (18.3%)		78 (95.1%)	4 (4.88%)	
Current fluid intake (mL)	500 [245–1000]	500 [262–1000]	0.476	500 [375–1000]	500 [200–750]	0.021 *	500 [300–1000]	250 [200–500]	0.014 *
Follow-up (%)			0.06			0.047			0.634
Nurse	52 (29.4%)	125 (70.6%)		149 (84.2%)	28 (15.8%)		167 (94.4%)	10 (5.65%)	
Physician	5 (41.7%)	7 (58.3%)		9 (75.0%)	3 (25.0%)		12 (100%)	0 (0.00%)	
Nursing technician	18 (48.6%)	19 (51.4%)		25 (67.6%)	12 (32.4%)		34 (91.9%)	3 (8.11%)	
Hemoglobin (g/dL)	12.9 ± 1.31	12.7 ± 1.16	0.425	12.7 ± 1.18	12.9 ± 1.37	0.584	12.7 ± 1.21	12.9 ± 1.38	0.644
Hematocrit (%)	40.0 [37.5–43.0]	40.0 [37.0–42.0]	0.527	40.0 [37.0–42.0]	40.0 [38.5–43.0]	0.245	40.0 [37.0–42.0]	40.0 [39.0–43.0]	0.364
Platelets (units/mm^3^)	200,000 [172,500–236,000]	200,000 [170,000–250,000]	0.427	200,000 [170,000–241,500]	196,000 [160,000–282,000]	0.678	200,000 [170,000–244,000]	190,000 [170,000–260,000]	0.854
Leukocytes (units/mm^3^)	6100 [5500–7700]	5950 [4900–7200]	0.137	6000 [5000–7450]	6150 [5400–6700]	0.693	6000 [5100–7400]	6000 [4850–6500]	0.37
Delay to care (days)	2.00 [1.00–4.50]	2.00 [1.00–4.00]	0.745	2.00 [1.00–5.00]	2.00 [1.00–3.00]	0.265	2.00 [1.00–4.00]	2.00 [2.00–3.00]	0.869
Fever (%)			0.042 *			0.62			1
No	5 (71.4%)	2 (28.6%)		5 (71.4%)	2 (28.6%)		7 (100%)	0 (0.00%)	
Yes	70 (32.0%)	149 (68.0%)		178 (81.3%)	41 (18.7%)		206 (94.1%)	13 (5.94%)	
Headache (%)			0.934			0.598			0.076
No	16 (34.8%)	30 (65.2%)		39 (84.8%)	7 (15.2%)		46 (100%)	0 (0.00%)	
Yes	59 (32.8%)	121 (67.2%)		144 (80.0%)	36 (20.0%)		167 (92.8%)	13 (7.22%)	
Myalgias (%)			0.7			0.559			0.556
No	26 (35.6%)	47 (64.4%)		57 (78.1%)	16 (21.9%)		70 (95.9%)	3 (4.11%)	
Yes	49 (32.0%)	104 (68.0%)		126 (82.4%)	27 (17.6%)		143 (93.5%)	10 (6.54%)	
Arthralgia (%)			0.63			0.743			1
No	45 (34.9%)	84 (65.1%)		103 (79.8%)	26 (20.2%)		122 (94.6%)	7 (5.43%)	
Yes	30 (30.9%)	67 (69.1%)		80 (82.5%)	17 (17.5%)		91 (93.8%)	6 (6.19%)	
Bilateral polyarthralgia (%)			0.512			0.017			1
No	68 (32.4%)	142 (67.6%)		174 (82.9%)	36 (17.1%)		198 (94.3%)	12 (5.71%)	
Yes	7 (43.8%)	9 (56.2%)		9 (56.2%)	7 (43.8%)		15 (93.8%)	1 (6.25%)	
Retro-orbital pain (%)			0.668			0.549			0.543
No	54 (34.4%)	103 (65.6%)		125 (79.6%)	32 (20.4%)		149 (94.9%)	8 (5.10%)	
Yes	21 (30.4%)	48 (69.6%)		58 (84.1%)	11 (15.9%)		64 (92.8%)	5 (7.25%)	
Rash/exanthem (%)			1			1			1
No	72 (33.2%)	145 (66.8%)		175 (80.6%)	42 (19.4%)		204 (94.0%)	13 (5.99%)	
Yes	3 (33.3%)	6 (66.7%)		8 (88.9%)	1 (11.1%)		9 (100%)	0 (0.00%)	
Recent travel (%)			1			0.358			1
No	75 (33.3%)	150 (66.7%)		175 (80.3%)	43 (19.7%)		205 (94.0%)	13 (5.96%)	
Yes	0 (0.00%)	1 (100%)		8 (100%)	0 (0.00%)		8 (100%)	0 (0.00%)	
Low back pain (%)			0.689			0.733			0.522
No	53 (32.1%)	112 (67.9%)		135 (81.8%)	30 (18.2%)		154 (93.3%)	11 (6.67%)	
Yes	22 (36.1%)	39 (63.9%)		48 (78.7%)	13 (21.3%)		59 (96.7%)	2 (3.28%)	
Conjunctivitis (%)			1			1			1
No	74 (33.2%)	149 (66.8%)		180 (80.7%)	43 (19.3%)		210 (94.2%)	13 (5.83%)	
Yes	1 (33.3%)	2 (66.7%)		3 (100%)	0 (0.00%)		3 (100%)	0 (0.00%)	
Nausea (%)			0.706			0.558			0.138
No	45 (31.9%)	96 (68.1%)		112 (79.4%)	29 (20.6%)		130 (92.2%)	11 (7.80%)	
Yes	30 (35.3%)	55 (64.7%)		71 (83.5%)	14 (16.5%)		83 (97.6%)	2 (2.35%)	
Vomiting (%)			0.826			0.219			0.232
No	52 (34.0%)	101 (66.0%)		120 (78.4%)	33 (21.6%)		142 (92.8%)	11 (7.19%)	
Yes	23 (31.5%)	50 (68.5%)		63 (86.3%)	10 (13.7%)		71 (97.3%)	2 (2.74%)	
Diarrhea (%)			0.289			0.805			0.451
No	66 (34.9%)	123 (65.1%)		152 (80.4%)	37 (19.6%)		179 (94.7%)	10 (5.29%)	
Yes	9 (24.3%)	28 (75.7%)		31 (83.8%)	6 (16.2%)		34 (91.9%)	3 (8.11%)	
Altered mental status (%)			1			1			1
No	75 (33.3%)	150 (66.7%)		182 (80.9%)	43 (19.1%)		212 (94.2%)	13 (5.78%)	
Yes	0 (0.00%)	1 (100%)		1 (100%)	0 (0.00%)		1 (100%)	0 (0.00%)	
Tachycardia (%)			1			0.001			0.001 **
No	74 (33.3%)	148 (66.7%)		183 (82.4%)	39 (17.6%)		212 (95.5%)	10 (4.50%)	
Yes	1 (25.0%)	3 (75.0%)		0 (0.00%)	4 (100%)		1 (25.0%)	3 (75.0%)	

Note. Variables are presented as mean ± SD, median [interquartile range], or absolute and relative frequency (%). * *p* < 0.05 or ** *p* < 0.01, statistically significant by Mann–Whitney U, chi-square, or Fisher’s exact test.

**Table 3:** Poisson regression models of clinical factors associated with dengue, leptospirosis, and coinfection.

Infection/Variable	cPR	95% CI	*p*-Value
Leptospira–dengue coinfection			
Temperature (°C)	1.75	1.05–3.01	0.036
Dengue			
Tachycardia (yes vs. no)	5.69	1.95–13.07	<0.001
Bilateral polyarthralgia (yes vs. no)	2.55	1.14–5.04	0.012
Mucosal bleeding/ecchymosis (yes vs. no)	3.31	0.94–8.37	0.027
Temperature (°C)	1.61	1.23–2.12	0.001
Leptospirosis			
Male sex (vs. female)	0.78	0.65–0.94	0.01
Fever (yes vs. no)	2.38	1.17–6.03	0.035

Note: cPR = crude prevalence ratio estimated using Poisson regression models with robust variance. Reference categories were absence of the sign/symptom for dichotomous variables and female sex for the sex variable.

## Data Availability

The original contributions presented in this study are included in the article. Further inquiries can be directed to the corresponding authors.

## References

[1] Orozco Sotomayor S.P., Rojas Pua N.J., Trujillo González L.M., Vanegas Martínez T.P., Ureche Gámez E.C., Díaz Burgos S.C., Bravo Solarte D.L., Atilano Macias D.J., Caballero Morales L.A. (2024). Dengue y Leptospira: Fisiopatología y Coincidencias Clínicas. Cienc. Lat. Rev. Científica Multidiscip..

[2] World Health Organization (2023). Dengue—Global Situation (Disease Outbreak News No. 2023-DON498).

[3] Schneider M.C., Leonel D.G., Hamrick P.N., de Caldas E.P., Velásquez R.T., Paez F.A.M., Arrebato J.C.G., Gerger A., Pereira M.M., Aldighieri S. (2017). Leptospirosis in Latin America: Exploring the first set of regional data. Rev. Panam. De Salud Publica Pan Am. J. Public Health.

[4] Centro Nacional de Epidemiología (2024). Número de Casos de Leptospirosis, Perú 2019–2024. https://www.dge.gob.pe/portal/docs/vigilancia/sala/2024/SE45/leptospirosis.pdf.

[5] Gomez M.D.N., Marín-Macedo H., Seminario-Vilca R. (2021). Dengue with signs of alarm and leptospirosis in a pediatric patient with COVID-19. Rev. De La Fac. De Med. Humana.

[6] Veena R., Kumar K.V., Swathi M., Bokade P., Pal A., SowjanyaKumari S., Arun Y., Devaraj S., Jagadeesha K., Padma M. (2024). Epidemiological analysis of leptospirosis, dengue, and Co-infection rates among febrile illness cases in Dakshina Kannada, Karnataka. Indian J. Med. Microbiol..

[7] Boey K., Shiokawa K., Rajeev S. (2019). Leptospira infection in rats: A literature review of global prevalence and distribution. PLoS Neglected Trop. Dis..

[8] Centro Para el Control y la Prevención de Enfermedades de Estados Unidos (2023). Centros Para el Control y la Prevención de Enfermedades (CDC): Proteger a la Población de las Amenazas Para la Salud. https://www.paho.org/es/alianzas/centros-para-control-prevencion-enfermedades-cdc-proteger-poblacion-amenazas-para-salud.

[9] Costa F., E Hagan J., Calcagno J., Kane M., Torgerson P., Martinez-Silveira M.S., Stein C., Abela-Ridder B., I Ko A. (2015). Global Morbidity and Mortality of Leptospirosis: A Systematic Review. PLoS Neglected Trop. Dis..

[10] Organización Panamericana de la Salud (2018). Estrategia de Gestión Integrada Para la Prevención y el Control del Dengue. https://www.paho.org/es/temas/dengue/estrategia-gestion-integrada-para-prevencion-control-dengue.

[11] Loong S.K., Abd-Majid M.A., Teoh B.T., Cheh M.J., Khor C.S., Chao C.C., Khoo J.J., AbuBakar S. (2022). Leptospirosis among Dengue-Negative Febrile Patients in Selangor, Malaysia. Am. J. Trop. Med. Hyg..

[12] Fornazari F., Richini-Pereira V.B., Joaquim S.F., Nachtigall P.G., Langoni H. (2021). Leptospirosis diagnosis among patients suspected of dengue fever in Brazil. J. Venom. Anim. Toxins Incl. Trop. Dis..

[13] Asaduzzaman M., Karmaker L., Rahman A., Rahman M.S., Awaul M.A., Chakraborty S.R. (2024). Dengue and leptospirosis coinfection: A case series. J. Med. Case Rep..

[14] Vagha K., Uke P., Varma A., Javvaji C.K., Malik A., Murhekar S., Sr A.V. (2024). Concurrently Affected by Dengue and Hepatitis A: Exploring the Intricacies of Co-infection in a Comprehensive Case Series. Cureus.

[15] Erickson T.B., Brooks J., Nilles E.J., Pham P.N., Vinck P. (2019). Environmental health effects attributed to toxic and infectious agents following hurricanes, cyclones, flash floods and major hydrometeorological events. J. Toxicol. Environ. Health Part B Crit. Rev..

[16] Ferreira-De-Lima V.H., Lima-Camara T.N. (2018). Natural vertical transmission of dengue virus in Aedes aegypti and Aedes albopictus: A systematic review. Parasites Vectors.

[17] Hacker K.P., Sacramento G.A., Cruz J.S., de Oliveira D., Nery N., Lindow J.C., Carvalho M., Hagan J., Diggle P.J., Begon M. (2020). Influence of rainfall on leptospira infection and disease in a tropical urban setting, Brazil. Emerg. Infect. Dis..

[18] Avalos C.A., Cristaldi M.A., Mendicino D.A., Previtali A. (2023). Differences in the association of dengue and leptospirosis incidences with respect to socio-sanitary vulnerability in the city of Santa Fe, Argentina. Soc. Med..

[19] Zavala-Velázquez J.E. (1998). Leptospirosis anictérica en un brote epiémico de dengue en la Península de Yucatán. Rev. Biomed..

[20] LaRocque R.C., Breiman R.F., Ari M.D., Morey R.E., Janan F.A., Hayes J.M., Hossain M.A., Brooks W.A., Levett P.N. (2005). Leptospirosis during dengue outbreak, Bangladesh. Emerg. Infect. Dis..

[21] Brown M.G., E Vickers I., Salas R.A., Smikle M.F. (2010). Leptospirosis in suspected cases of dengue in Jamaica, 2002–2007. Trop. Dr..

[22] Cabello M.A., Cabral M.B., Samudio M., Páez M., Jiménez R., Arce M., Leguizamón M.A. (2010). Dengue and leptospirosis share the same ecological niche in Carmen del Paraná (Itapua), a riverside locality. Mem. Inst. Investig. Cienc. Salud.

[23] Crociati L., Onofre H. (2010). Leptospirosis and dengue co-infection in a Brazilian Amazon patient. Rev. Panamazonica Saude.

[24] Dircio Montes Sergio A., González Figueroa E., María Saadia V.G., Elizabeth S.H., Beatriz R.S., Altuzar Aguilar Víctor M., Navarrete Espinosa J. (2012). Leptospirosis prevalence in patients with initial diagnosis of dengue. J. Trop. Med..

[25] Suppiah J., Chan S.-Y., Ng M.-W., Khaw Y.-S., Ching S.-M., Mat-Nor L.A., Ahmad-Najimudin N.A., Chee H.-Y. (2017). Clinical predictors of dengue fever co-infected with leptospirosis among patients admitted for dengue fever—A pilot study. J. Biomed. Sci..

[26] Musso D., La Scola B. (2013). Laboratory diagnosis of leptospirosis: A challenge. J. Microbiol. Immunol. Infect..

[27] Le Turnier P., Mosnier E., Schaub R., Bourhy P., Jolivet A., Cropet C., Villemant N., Trombert-Paolantoni S., Berlioz-Arthaud A., Nacher M. (2018). Epidemiology of human leptospirosis in French Guiana (2007–2014): A retrospective study. Am. J. Trop. Med. Hyg..

[28] Goswami R.P., Basu A., Tripathi S.K., Chakrabarti S., Chattopadhyay I. (2014). Predictors of mortality in leptospirosis: An observational study from two hospitals in Kolkata, eastern India. Trans. R. Soc. Trop. Med. Hyg..

[29] Núñez-Garbín A., Espinoza-Figueroa J., Sihuincha-Maldonado M., Suarez-Ognio L. (2015). Coinfección por dengue y leptospirosis en una niña de la Amazonía Peruana. Rev. Peru Med. Exp. Salud Publica.

[30] Rodríguez R., Gonzáles A., Palacios A. (2014). Leptospirosis in present environment. Rev. Electrónica.

[31] Sunil-Chandra N.P., Clement J., Maes P., De Silva H.J., Van Esbroeck M., Van Ranst M. (2015). Concomitant leptospirosis-hantavirus co-infection in acute patients hospitalized in Sri Lanka: Implications for a potentially worldwide underestimated problem. Epidemiol. Infect..

[32] Kumar P., Kumar R., Singh M.K., Ahmed B. (2024). Detection of Plasma, Platelets, Hemoglobin in Blood Sample of Dengue Malaria Based on Surface Plasmon Resonance Biosensor Using Black Phosphorus: A Numerical Analysis. Plasmonics.

[33] del Valle-Mendoza J., Palomares-Reyes C., Carrillo-Ng H., Tarazona-Castro Y., Kym S., Aguilar-Luis M.A., del Valle L.J., Aquino-Ortega R., Martins-Luna J., Peña-Tuesta I. (2021). Leptospirosis in febrile patients with suspected diagnosis of dengue fever. BMC Res. Notes.

[34] Vargas F. (2024). Síndrome Febril y los Casos Confirmados de Dengue en el Hospital Santa Rosa de Madre de Dios Durante el Periodo Noviembre 2021 a Abril 2022. Bachelo’s Thesis.

[35] Presidencia de la República del Perú (2024). Decreto Supremo N.° 005-2024-SA (Normas Legales N.° 31427).

[36] Palma Mezones T.N., Pincay Delgado K.M., Piguave Reyes J.M. (2023). Infecciones Vectoriales: Manifestaciones Clínicas Y Diagnóstico Diferencial. MQRInvestigar.

[37] Argimon J.M. (2019). Metodos de Investigación Clinica y Epidemiologica.

[38] Minsa (2024). Norma Técnica de Salud Para la Atención Integral de Pacientes con Dengue en el Perú. NTS N°211-MINSA/DGIESP. https://cdn.www.gob.pe/uploads/document/file/6007546/5323501-r-m-175-2024-minsa-y-nts-211-dgiesp.pdf?v=1709834791.

[39] Céspedes Z.M., Ormaeche M.M., Condori P., Balda J.L., Glenny A.M. (2003). Prevalencia de Leptospirosis y factores de riesgo en personas con antecedentes de fiebre en la Provincia de Manu, Madre de Dios, Perú. Rev. Peru Med. Exp. Salud Publica.

[40] Silva-Díaz H., Llatas-Cancino D.N., Campos-Sánchez M.J., Aguilar-Gamboa F.R., Mera-Villasis K.M., Valderrama-Ayén M.Y. (2015). Frecuencia de leptospirosis y características socio-demográficas en pacientes febriles del norte del Perú. Rev. Chil. De Infectol..

[41] Alarcón-Villaverde J., Romani-Romani F., Tejada R., Wong-Chero P., Céspedes_Zambrano M. (2014). Seroprevalencia de leptospirosis y características sociadas en agricultores de arroz de una región tropical del Perú. Rev. Peru Med. Exp. Salud Publica.

[42] Rafizah A.N., Aziah B., Azwany Y., Imran M.K., Rusli A.M., Nazri S.M., Nikman A.M., Nabilah I., Asma’ H.S., Zahiruddin W. (2013). A hospital-based study on seroprevalence of leptospirosis among febrile cases in northeastern Malaysia. Int. J. Infect. Dis..

[43] Dhanashree B., Shenoy S. (2021). Seropositivity for dengue and leptospira igm among patients with acute febrile illness: An indicator of co-infection?. Germs.

[44] Pérez-Gutiérrez N., Amador-León P.A. (2021). Dengue: News and standards in clinical approach. Acta Colomb. De Cuid. Intensivo.

[45] Bolaños-Díaz M.C., López-Delgado D., Arango-Luque A., Polanco-Pasaje J.E. (2025). Factores asociados a dengue grave en pacientes del departamento del Cauca-Colombia 2015–2021. Iatreia.

[46] Guevara K., López M., Marroquín J., Sotz J., Tello del Valle E. (2025). Incidencia y Caracterización Epidemiológica del Dengue en la Población en General que Acude al Hospital IGSS de Huehuetenango, Ubicado en el Departamento de Huehuetenango, Durante el Período de Julio a Diciembre 2024. PhD. Thesis.

[47] Negreiros G.C.L., Vásquez S.R., Hipólito J.V.V. (2024). Clinical characteristics and epidemiological situation of dengue fever in Peru: A systematic review. Rev. Del Cuerpo Med. Hosp. Nac. Almanzor Aguinaga Asenjo.

[48] Valdivia D. (2021). Nivel de Asociación Entre Trombocitopenia y las Manifestaciones Clínicas del Dengue en Pacientes Atendidos en un Hospital II-I en la Ciudad de Ica Entre Enero del 2018 y Junio del 2020. Bachelor’s Thesis.

